# Editorial: Well-being and cognitive science in higher education: measures and intervention

**DOI:** 10.3389/fpsyg.2026.1847789

**Published:** 2026-06-01

**Authors:** Prisla Ücker Calvetti, Sandra Carvalho, Jorge Leite

**Affiliations:** 1 Moinhos de Vento Faculty of Health Sciences, Moinhos de Vento Hospital, Porto Alegre, Brazil; 2 Federal University of Health Sciences of Porto Alegre [Federal University of Health Sciences of Porto Alegre (UFCSPA), National Council for Scientific and Technological Development (CNPq)], Porto Alegre, Brazil; 3 Psychological Neuroscience Laboratory, Psychology Research Center (CIPsi), School of Psychology, University of Minho, Braga, Portugal; 4 Portucalense Institute for Human Development, Portucalense University, Porto, Portugal

**Keywords:** cognitive psychology, health promotion, health psychology, higher education, wellbeing

Higher education institutions are increasingly challenged to address not only academic achievement but also the psychological wellbeing and socio-cognitive development of students, particularly in light of rising levels of stress, anxiety, and academic pressure in university populations (Fang et al., 2025; Pascoe et al., 2020). Recent research highlights the complex interplay between cognitive processes, psychosocial resources, and institutional practices in shaping students' wellbeing and academic functioning, emphasizing the need for integrated and systemic approaches.

This Research Topic, *Well-Being and cognitive science in higher education: measures and intervention*, brings together 19 contributions that explore these dynamics through diverse methodological approaches, including empirical studies, qualitative analyses, systematic reviews, and intervention research. Several contributions focus on determinants of wellbeing and psychological functioning in higher education contexts. These studies examine how individual and contextual factors—such as social status, motivation, academic demands, and institutional environments—shape students' cognitive and emotional experiences. Recent evidence suggests that student wellbeing is strongly influenced by the balance between academic demands and coping resources, as well as by access to institutional and social support systems.

Another group of articles investigates self-regulatory and cognitive mechanisms that support academic functioning and wellbeing. Contemporary research has reinforced the central role of self-regulated learning (SRL), demonstrating its association with motivation, academic performance, and psychological wellbeing (Rodríguez et al., 2022). In particular, recent studies show that self-regulation and mindfulness positively predict academic self-efficacy, with mental wellbeing acting as a key mediating mechanism. These findings reinforce the importance of metacognitive and emotional regulation strategies in higher education.

In addition, emerging research using learning analytics and longitudinal designs has demonstrated that student engagement and self-regulation evolve dynamically over time, with distinct behavioral patterns associated with academic success and persistence (Saqr and López-Pernas, 2024). These findings suggest that cognitive and behavioral processes should be understood as complex, time-dependent systems rather than static traits.

In addition to identifying determinants of wellbeing, several contributions explore innovative educational and psychosocial interventions designed to enhance interpersonal skills, resilience, and mental health among students. Recent intervention studies highlight the effectiveness of feedback-rich, psychologically safe learning environments in reducing anxiety and promoting self-regulation and engagement (McGuire, 2026). Similarly, advances in game-based learning, immersive environments, and AI-supported education have demonstrated promising results in fostering experiential learning and cognitive engagement (Makransky and Petersen, 2021; Plass et al., 2020; Shareefa et al., 2025; Abdel Hadi and Al-Quraan, 2025; see also recent AI-supported SRL research).

Complementing these intervention approaches, recent work has emphasized the role of mindfulness, self-efficacy, and emotional regulation in supporting both academic performance and psychological wellbeing. Evidence suggests that these constructs interact in a mutually reinforcing manner, contributing to adaptive learning behaviors and improved mental health outcomes.

Taken together, the contributions in this Research Topic underscore several key insights. First, student wellbeing is a multidimensional construct shaped by cognitive, emotional, social, and institutional factors. Second, self-regulation, mindfulness, and cognitive skills play a central role in enabling students to navigate academic demands effectively. Third, innovative pedagogical and technological interventions—including feedback-based models, experiential learning, and AI-supported systems—offer promising pathways for enhancing both learning and wellbeing.

Future research should prioritize longitudinal, data-driven, and intervention-based designs to better understand causal mechanisms linking cognitive processes, educational practices, and wellbeing outcomes. Additionally, interdisciplinary and cross-cultural approaches will be essential for developing scalable and context-sensitive strategies capable of addressing the evolving challenges faced by higher education institutions worldwide, in line with broader international educational policy frameworks (OECD, 2021).
